# Synergetic effects of *Ulva lactuca* and *Pterocladiella capillacea* on the multidrug-resistant *Klebsiella pneumoniae*

**DOI:** 10.1186/s12866-025-04102-4

**Published:** 2025-06-26

**Authors:** Rania M. Mahmoud, Gehad M. Khedr, Reda M. Taha, Asmaa A. Adawy

**Affiliations:** Botany Department, Faculty of Science, Fayoum University, Fayoum, Egypt

**Keywords:** Synergetic effects, *Ulva lactuca*, *Pterocladiella capillacea*, *Klebsiella pneumoniae*, Drug resistant

## Abstract

**Background:**

The potential of marine algae as a source of antibacterial chemicals has been very promising. Numerous bioactive compounds produced by these organisms can fight off harmful microorganisms. Two marine algae (Chlorophyta: *Ulva lactuca*, Rhodophyta: *Pterocladiella capillacea*) were screened for their antibacterial activity against distinct multidrug-resistant bacteria *Klebsiella pneumoniae*. This efficiency is ascribed to the bioactive substances found in these algae, which have the ability to stop the growth of this harmful bacteria. *Pterocladiella capillacea* is a viable target for the development of novel antibacterial medications since its chemicals function by interfering with bacterial processes.

**Results:**

A mixture of (*U. lactuca + P. capillacea.* + Nitrofurantoin) increased the inhibition zone on *K. pneumoniae* by 100%. Our docking findings demonstrated that benzo[h]quinoline, 2, 4-dimethyl-, and 4, 4’-Bis [4-methyl-2-pyrimidylsulfamido] terephthalanilide have a high affinity (-10 and − 11 respectively) to dock with the cell wall proteins of *Klebsiella pneumoniae*. It can cause cell lysis and death by interfering with the integrity of bacterial cell walls. Furthermore, it may disrupt DNA replication and vital bacterial enzymes. The molecule 4, 4’-Bis [4-methyl-2-pyrimidylsulfamido] terephthalanilide is a member of the sulfonamide class, which is well-known for its broad-spectrum antibacterial properties. This compound’s capacity to prevent bacteria from synthesizing folic acid is the main source of its antibacterial action. Bacterial cell division and DNA synthesis depend on folic acid. The substance successfully stops bacterial growth and multiplication by blocking this pathway.

**Conclusion:**

These two substances are interesting candidates for the development of novel antimicrobial drugs due to their efficacy against strains of bacteria that are resistant to antibiotics.

**Supplementary Information:**

The online version contains supplementary material available at 10.1186/s12866-025-04102-4.

## Introduction

Antibiotics were first discovered during the golden age in the 1940 s to 1970 s, and they are compounds that inhibit bacterial cell wall synthesis, DNA replication and protein synthesis [1, 2]. Antibiotic resistance against a wide spectrum of pathogens and bacteria can result from the irrational use of antibiotics. The benefit of antibiotics is being threatened by the development of resistance; moreover, antibiotic resistance is a major worldwide issue [3, 4]. Because of the development of antibiotic resistance, antibiotic alternatives are urgently required [1]. It has been demonstrated that using natural ingredients to create medications to treat bacterial infections is quite effective [5]. Molds and plants produce antibiotic substances; therefore, the ancient Egyptians used them to treat infections [6]. Recently, there has been a successful investigation into the possible use of bioactive compounds obtained from the marine environment as viable chemo preventive agents with few side effects and proven safety [7]. Algae are photosynthetic organisms ranging from prokaryotic cyanobacteria to eukaryotic that are able to grow even in extreme conditions [8, 9]. Besides, algae are thallophytes (plants without true roots, stems, or leaves) with chlorophyll a as their major photosynthetic pigment and are autotrophs [10]. Macroalgae, also referred to as seaweeds, are enormous, eukaryotic, aquatic photosynthetic plants that fall into three categories and are visible without a microscope: Rhodophyta, Phaeophyta, and Chlorophyta types [11–14] examined a number of studies on the biological activity of seaweeds; furthermore, other researchers have found that chemicals originating from marine algae display a variety of biological activities [15, 16]. Marine macroalgae are the most fascinating type of algae, due to their wide range of biological activity, including antimicrobial properties [17, 18], anti-oxidative [19, 20], anti-coagulant [21, 22], antifouling [23, 24], anticancer [25, 26] and anti-microbial [27, 28]. Algae has been used to treat a number of chronic illnesses, including diabetes mellitus, cancer, cardiovascular disease, osteoporosis, and neurological diseases [29]. Besides, algae is a beneficial and alternative source with many pharmacological, biofuel production, and biological activities [30, 31]. Among Marine macroalgae: Because *Ulva* species are the most edible seaweeds and are widespread in coastal benthic locations worldwide, they represent an inexpensive and plentiful source of biomass [32–34].

*Klebsiella* species are Gram-negative, nonmotile, usually encapsulated rod-shaped bacteria of the family *Enterobacteriaceae* [35]. *Klebsiella* species can cause a variety of nosocomial infections in humans; moreover, *Klebsiella* primarily attacks immunocompromised individuals who are hospitalized and who have severe underlying diseases [36]. *Klebsiella pneumoniae* can cause infections including pneumonia, urinary tract infections, bloodstream infections, Pyogenic liver abscess and lower gastrointestinal tract diseases [37–42]. The increasingly antibiotic-resistant *K. pneumoniae* infection is becoming a major health threat [43]. This global issue requires the development of new sources of accessible, essential, naturally produced compounds in order to defeat diseases such as algae [44]. Synergism between antibiotics and algal extracts can have either synergistic or antagonistic effects [45]. In addition, a synergistic effect was observed for the combination of algae or cyanobacteria with algae [46, 47]. Hence, the purpose of this study is, isolation of a pathogenic multidrug resistant bacteria (*K. pneumoniae*) from patients and finding an effective bioactive compound from marine algae which can be use as antibiotics against it.

## Materials and methods

### Collection of algae

In this study, 2 species of seaweeds were collected from Rocky Bay of Abu Qir, Alexandria coast, Egypt (N 31° 19` E 030° 03`) during April 2021. The collected seaweeds was preserved for identification and all the seaweeds were identified following [48–50].

### Preparation of algal extract

After collection, the samples were washed with fresh water several times to remove salts and debris, then air dried. The extraction of powdered algal samples was done using absolute ethanol and methanol according to [44].

### Morphological, biochemical and molecular identification of bacterial isolates

Bacterial strains were collected from Al**-**Fayoum General Hospital, Fayoum Governorate, Egypt. *K. pneumoniae* were isolated from urine and sputum samples as gram-negative bacteria. All samples were cultured primarily in nutrient broth at 37 °C for 18–24 h, then sub-cultured on to the MacConkey agar, Nutrient agar, and Blood agar by streak plate method [51]. Based on the colonies morphology (shape, size, surface, texture, color etc.) and biochemical tests according to Bergey’s manual [52], the isolated bacteria were identified prima. Coagulase test done according to [53], Catalase test done according to [54], Oxidase test done according to [55], Urease test according to [56], Methyl Red Voges -Proskauer (V-P) test and Indole test done according to [57], Identification of bacterial isolates [58].

Molecular identification was done by the amplification of the 16 S rDNA gene, using forward primer (F1; AGA GTT TGA TCC TGG CTC AG) and reverse primer (R1; GGT TAC CTT GTT ACG ACT T). Sequencing of the amplified fragments was performed at GATC Biotech, Constance, Germany. The NCBI Data Base was used to align DNA sequences (www.ncbi.nlm.nlh.gov). The identified strain was submitted in the gene bank under the accession number PP439638.1.

### Antibiotic sensitivity test and minimum inhibitory concentration

The antibacterial susceptibility of isolated bacterial strains was evaluated according to [59]. Ten antibiotics disc were tested on nutrient agar plates, and the diameter of the inhibition zones was measured in millimeters after 18–24 h of incubation at 35± 2 °C [60]. The antibacterial activities of investigated algal species were determined by well diffusion method proposed by [61]. Additionaly, the Minimal Inhibitory Concentration (MIC) values of the algal extracts were determined using the agar dilution method, with bacterial growth observed after 24 h of incubationto identify the lowest concentration that inhibited visivle growth [62].

### Synergetic effect

The synergistic effect (SE) between ethanolic extracts of *U. lactuca* and *P.capillacea*, either individually or in combination with the antibiotic nitrofurantoin (300 µg), against *Klebsiella pneumoniae* was evaluated according to the method described by [63]. A 100 µL aliquot of the activated *K. pneumoniae* culture was evenly spread onto nutrient agar plates using a sterile swab. Eight wells (6 mm in diameter) were aseptically punched into each plate. Each well was loaded with 100 µL of the corresponding treatment (500 mg/ml): *either U. lactuca* extract, *P. capillacea* extract, nitrofurantoin (300 µg) alone, or their combinations. Plates were incubated at 37 °C for 24 h. Following incubation, the diameters of the zones of inhibition (ZOI) around each well were measured in millimeters. The percentage of synergistic effect was calculated using the following equation:

$$\:\text{S}\text{E}=\frac{B-A}{A}\times\:100$$

Where A is antibiotic ZOI and B is antibiotic and/or *algal* extract ZOI.

### Gas chromatography-mass spectrometry (GC–MS)

The crude ethanolic extract [64] and methanolic extract [65] of tested algae, were subjected to GC–MS analysis for determination of bioactive compounds. The bioactive components of algal extracts were determined using GC-MS (Thermo Scientific TRACE 1310 Gas Chromatograph attached with ISQ LT single quadrupole Mass Spectrometer).

### Molecular docking

Molecular docking studies were performed using AutoDock Vina [66] to forecast the compounds’ affinities and ways of interacting with every protein. The anticipated binding locations served as the centre of the docking grid boxes. For the docking calculations, the default scoring algorithm was employed, and the exhaustiveness value was set to 8.

### Visualization and analysis

The docked complexes were visualized and analyzed using BIOVIA Discovery Studio Visualizer 2020 [67]. The binding affinities (ΔG values) and intermolecular interactions, including hydrogen bonds and hydrophobic interactions, were analyzed and reported.

### In silico toxicity test

The most effective compounds’ cytotoxicity against *K. pneumoniae* was tested using https://tox.charite.de/protox3/index.php?site=home.

## Results

### Characters of pathogenic bacteria *K. pneumoniae*

Bacterial isolates were confirmed morphologically by their growth on solid selective medium and investigated microscopically by gram staining and some biochemical tests (Table S1). *K. pneumoniae* isolate was gram negative rods. Appear On Blood Agar as Dome-shaped and greyish white (Figure S1A) and On MacConkey agar as pink colored (Figure S1B).

### Phylogeny of *K. pneumoniae*

**Fig. 1:**
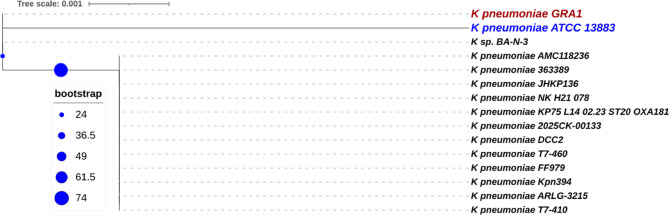
Phylogenetic tree of partial 16s rRNA showing the phylogenetic position of against *K. pneumoniae* (GRA1) (written in brown) compared with other related *K. pneumoniae* from the Gene Bank, including the most common reference strain ATCC 13,883(witten in blue). Bootstrap values for nodes are shown in the tree

Based on aligned 16S rRNA sequences from 15 *Klebsiella* strains, the IQ-TREE software was used to create the phylogenetic tree displayed above using a maximum likelihood (ML) strategy (Fig. 1). The strength of individual clades is indicated by bootstrap support values, which are shown as blue circles of increasing size. The great degree of sequence similarity across the examined species is highlighted by the scale bar, which represents a branch length of 0.001 substitutions per site.

A significant degree of phylogenetic similarity is suggested by the notable close clustering of *K. pneumoniae* GRA1 (in red) with the reference strain, *K. pneumoniae* ATCC 13,883 (in blue). This cluster also includes K. sp. BA-N-3, suggesting that it shares a tight evolutionary relationship with the reference strains.

### Sensitivity test of *K. pneumoniae*

Data in Figure S2 and Table 1 showed that sixteen antibiotics were used against *K. pneumoniae*, a sensitive isolate that showed resistant to eight antibiotics: Ampicillin/Sulbactam (20 µg), Ampicillin (10 µg), Amoxycillin (10 µg), Gentamicin (10 µg), Tobramycin (10 µg), Piperacillin/Tazobactam (110 µg), Ceftriaxone (30ug), Cefotaxime (30 µg). Moreover, Ampicillin/Sulbactam (20 µg), Ampicillin (10 µg), Amoxycillin (10 µg), Gentamicin (10 µg), Tobramycin (10 µg) showed (0 mm) zone. The isolate intermediate only to Cefoxitin (30 µg), Ceftazidime (30 µg). The isolate showed sensitive to six antibiotics: Cefepime (30 µg), Doxycycline (30 µg), Ofloxacin (5ug), Levofloxacin (5 µg), Doripenem (10 µg), Colistin (10 µg). this result indicates that *K. pneumoniae* is resistant to wide range of antibiotics, which consider a hazard alert for the shortage of available antibiotics against such bacteria.

**Table 1:** Inhibition zone (mm) by disc diffusion method of *K. pneumoniae*. S: susceptible, I: intermediate, R: resistant

Antibiotic class	Antimicrobial agent	Conc. (µg)	interpretive categories and zone diameter breakpoints nearest whole mm			*K. pneumoniae* Inhibition Zone (mm)by disc diffusion method
			S		R	
Penicillins	Ampicillin/Sulbactam (SAM)	20µg	≥15	12–14	≤11	-
	Ampicillin (AM)	10µg	≥ 17	14–16	≤ 13	-
	Amoxycillin (AML)	10µg	≥23	15–22	≤14	-
	Piperacillin/Tazobactam (TZP)	110µg	≥ 21	18–20	≤ 17	14
cephalosporin	Ceftriaxone (CRO)	30ug	≥ 23	20–22	≤ 19	17
	Cefotaxime (CTX)	30µg	≥26	23–25	≤22	20
	Ceftazidime (CAZ)	30µg	≥ 21	18–20	≤ 17	19
Beta-lactams	Cefepime (FEP)	30µg	≥18	15–17	≤14	18
Tetracyclines	Doxycycline (DO)	30µg	≥14	11–13	≤10	19
Fluoroquinolone	Ofloxacin (OFX)	5ug	≥16	13–15	≤12	17
	Levofloxacin (LEV)	5µg	≥17	14–16	≤13	20
carbapenem	Doripenem (DOR)	10µg	≥19	16–18	≤15	19
Lipopeptide	Colistin (CT)	10µg	≥13	10–12	≤9	15
aminoglycoside	Gentamicin (GM)	10µg	≥ 15	13–14	≤ 12	-
cephamycin	Cefoxitin (FOX)	30µg	≥18	15–17	≤14	17
aminoglycoside	Tobramycin (TOB)	10µg	≥ 15	13–14	≤ 12	-

### Identification of the studied algae

**Fig. 2:**
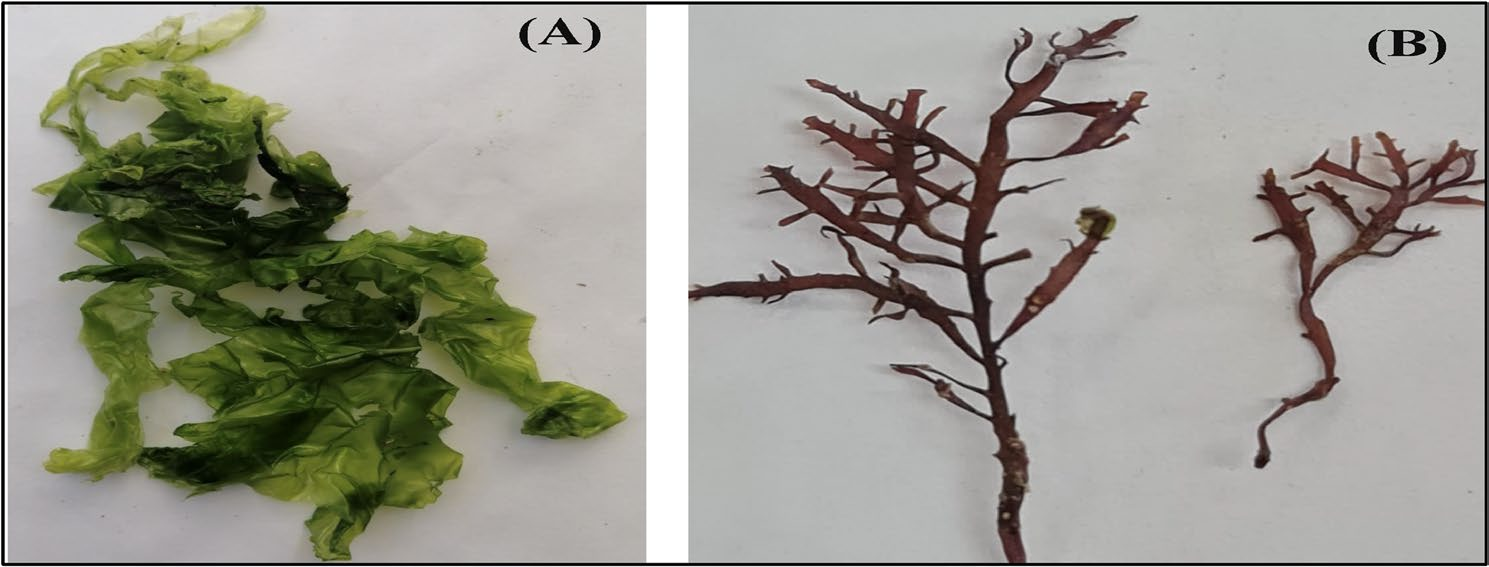
Isolated marine algal strains (**A**) Ulva Lactuca, **B** Pterocladiella capillacea

Two species of seaweeds were collected from Rocky Bay of Abu Qir, Alexandria coast, Egypt (N 31° 19` E 030° 03`) during April 2022, Chlorophyta: *U. lactuca* Fig. 2A and Rhodophyta: *P. capillacea* Fig. 2B.

### Minimal inhibitory concentration (MIC) determination

**Fig. 3:**
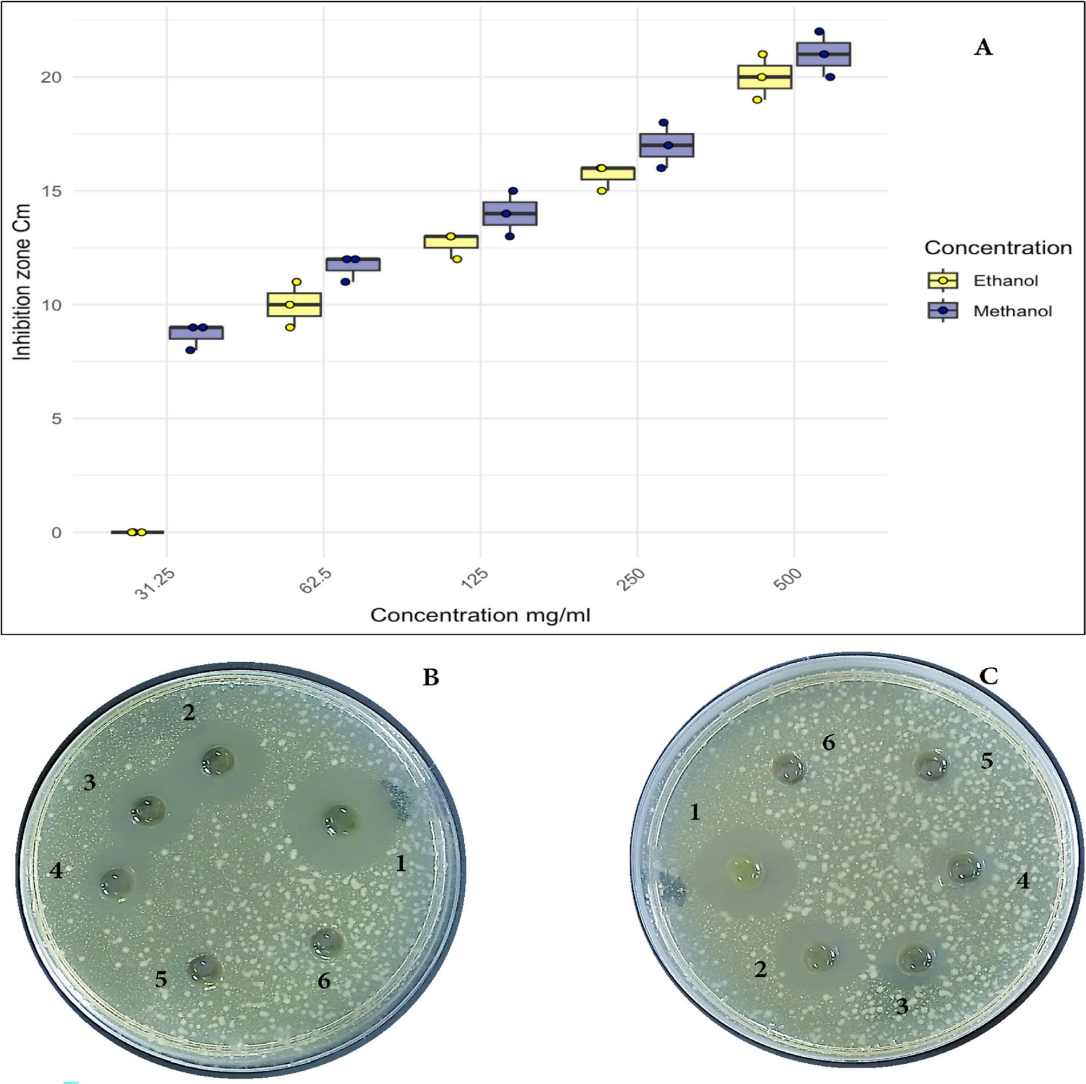
MIC value of *U. lactuca ***A** Box plot for the ethanolic and methanolic extract, panel **B** is the corresponding plate for ethanolic extracts and panel **C** is the corresponding plate for methanolic extract against *Klebsiella pneumoniae*; symbols on plates is for the used concentration 1: 500 mg/ml, 2: 250 mg/ml, 3: 125 mg/ml, 4: 62.5 mg/ml, 5: 31.25 mg/ml, 6: Negative control (5% DEMSO)

The MIC of the extracts from *U. lactuca*, and *P. capillacea*, were determined against *K. pneumoniae*, The MIC value was determined as the minimum concentration of algae capable to prevent bacterial growth. It was observed that *P. capillacea*, and *U. lactuca* ethanolic extracts have strong antimicrobial activity against *Klebsiella pneumoniae.* Although methanolic extract was more effective than ethanol, they both has a strong antimicrobial effect. The minimum inhibitory concentration of *U. Lactuca* was 31.25 mg/ml for methanolic extract however it was 62.5 mg/ml for ethanolic extract. This was indicated in Fig. 3 (A is a box plot for both ethanolic and methanolic extracts), (B the corresponding plate for ethanolic extract) and (C the corresponding plate for methanolic extract).

**Fig. 4:**
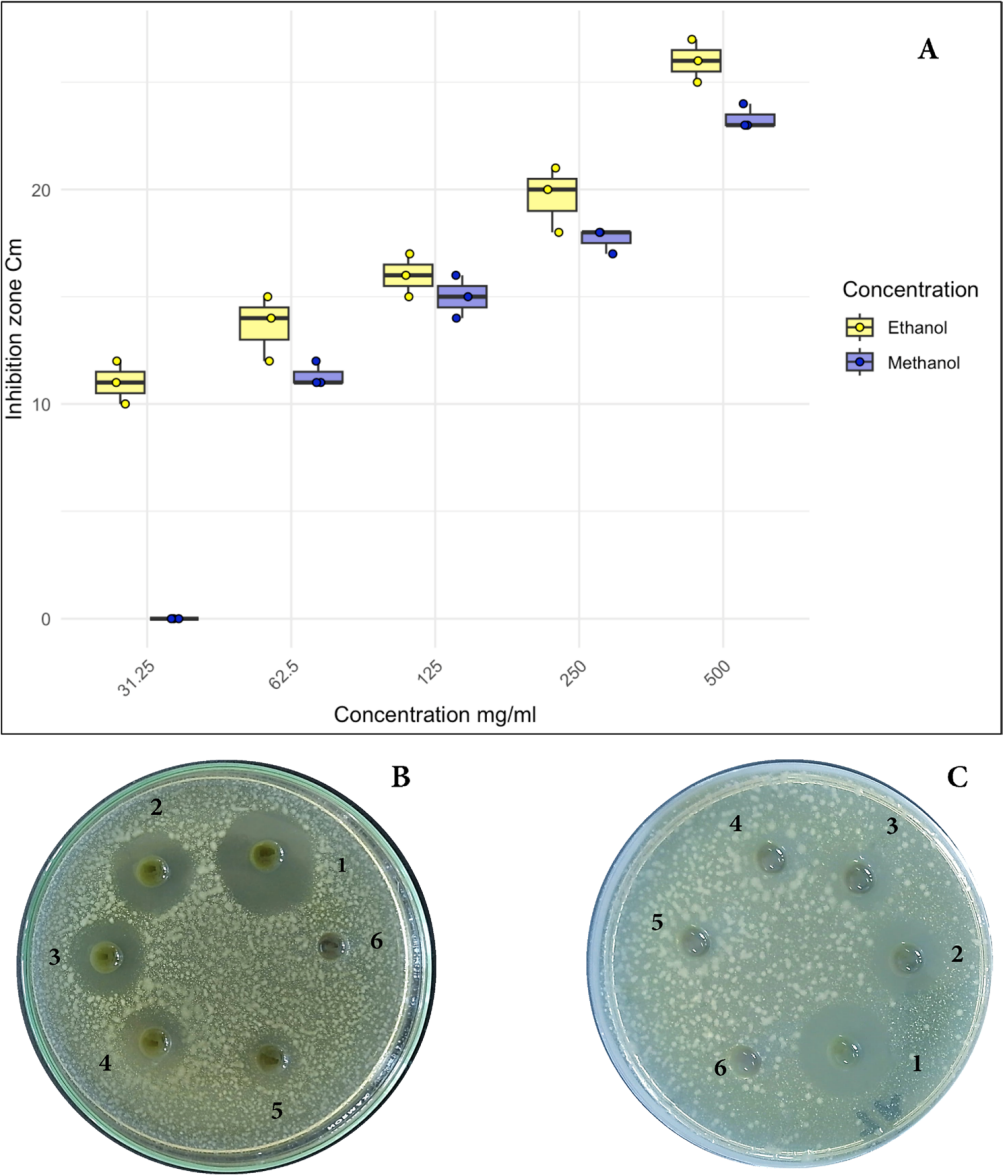
MIC value of *P. capillacea*, **A** Box plot for the ethanolic and methanolic extract, panel **B** is the corresponding plate for ethanolic extracts and panel **C** is the corresponding plate for methanolic extract against *K. pneumoniae*; symbols on plates is for the used concentration 1: 500 mg/ml, 2: 250 mg/ml, 3: 125 mg/ml, 4: 62.5 mg/ml, 5: 31.25 mg/ml, 6: Negative control (5% DEMSO)

A comparable result was obtained for *P. capillacea.* However, ethanolic extract was more effective than methanolic in this case. Where, the minimum inhibitory concentration of *P. capillacea* was 31.25 mg/ml for ethanolic extract however it was 62.5 mg/ml for methanolic extract. This was indicated in Fig. 4 (A is a box plot for both ethanolic and methanolic extracts), (B the corresponding plate for ethanolic extract) and (C the corresponding plate for methanolic extract).

### Synergistic effect

**Fig. 5:**
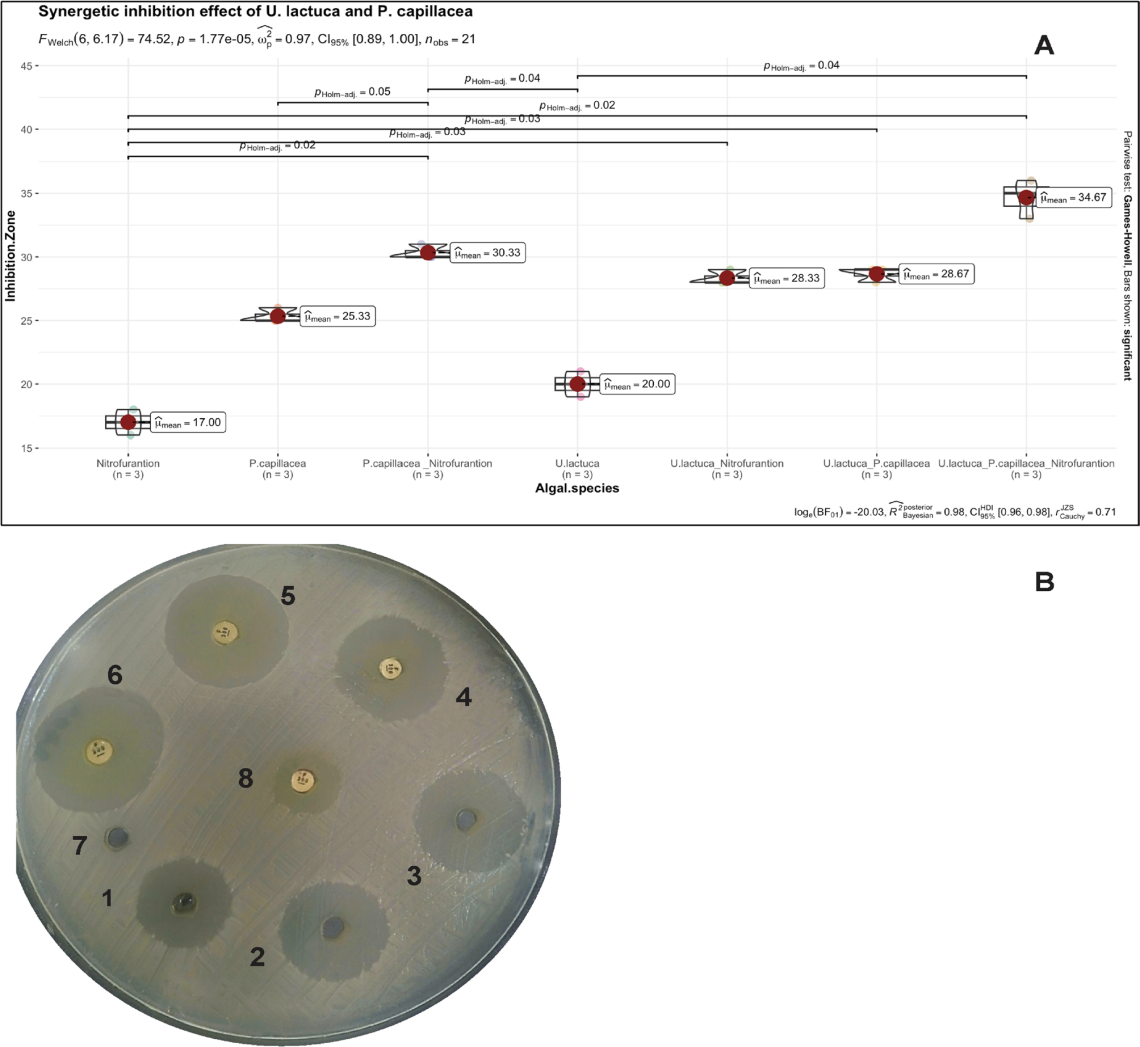
Synergetic inhibition effect of *Ulva lactuca* and *Pterocladiella capillacea* ethanolic extracts single or in combination with antibiotic (Nitrofurantion 300 μg) against *K. pneumoniae* strain GRA1; panel **A** is the box plot of the adjusted *p* value calculated. panel **B** is the corresponding plate for the synergesic effect; symbols on the agar plate indicate the used extract and/or antibiotics 1: *U. lactuca* ethanolic extract, 2: *Pterocladiella capillacea* ethanolic extract, 3: *U. lactuca* + *Pterocladiella capillacea*, 4: *U. lactuca* + Nitrofurantion, 5: *Pterocladiella capillacea*. + Nitrofurantion, 6: *U. lactuca* + *Pterocladiella capillacea*. + Nitrofurantion, 7: Negative control (5% DEMSO), 8: Positive control Nitrofurantion 300 μg

Synergetic inhibition effect of *K. pneumoniae* by the action of *U. lactuca* and *P. capillacea* ethanolic extracts and antibiotic (Nitrofurantoin 300 µg); (Fig. 5B) indicate that A: *U. lactuca* ethanolic extract at concentration 500 mg/ml increased the zone of inhibition by 18%, B: *P. capillacea* ethanolic extract at concentration 500 mg/ml increased the zone of inhibition by 48.80%, C: *U. lactuca* + *P. capillacea* ethanolic extracts at concentration 500 mg/ml increased the zone of inhibition 68.20%, D: *U. lactuca* + Nitrofurantoin at concentration 500 mg/ml increased the zone of inhibition by 66.47%, E: *P. capillacea.* + Nitrofurantoin at concentration 500 mg/ml increased the zone of inhibition by 78.20%, F: *U. lactuca* + *P. capillacea* + Nitrofurantoin at concentration 500 mg/ml increased the zone of inhibition by 100%, G: Negative control (5% DEMSO), H: Positive control Nitrofurantoin 300 µg. Although, the most effective mixture on *K. pneumoniae* was (*U. lactuca* + *P.capillacea.* + Nitrofurantoin) which increased the zone of inhibition by 100% but all different extracts single or in combination have a significant effect when compared to the antibiotic only Fig. 5A.

### Identification of bioactive compounds in tested algal extracts

**Fig. 6:**
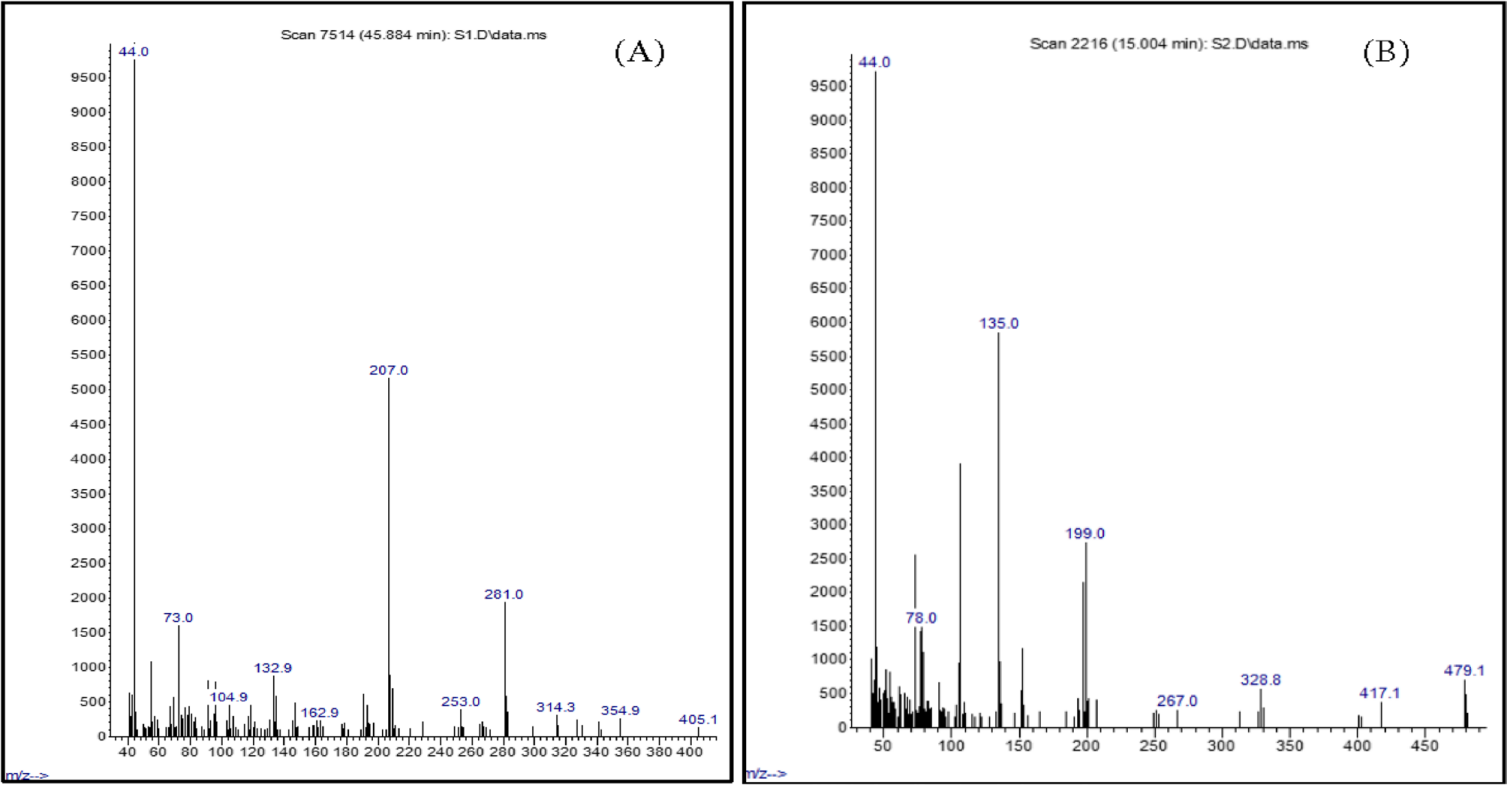
GC-Mas representation of bioactive compounds extracted from: **A** *U. lactuca*, **B*** P. capillacea*

The GC-MS analysis of *Ulva lactuca* and *P. capillacea* extracts (Fig. 6A and B; Tables S2 and S3, respectively) revealed the presence of multiple bioactive compounds, suggesting potential antibacterial activity. In *U. lactuca* extract (Table S2), the predominant compound identified was N-hexadecanoic acid (palmitic acid), comprising 73.57% of the total composition. Other notable compounds included sec-butyl N, N,P-trimethylphosphonamidate and 2-amino-1-(o-hydroxyphenyl)propane (4.77%), 3-methoxyamphetamine and norpseudoephedrine (4.38%), followed by allantoic acid, tetradecanoic acid, and pentadecanoic acid (2.83%), as well as benzo[h]quinoline, 2,4-dimethyl-, 2-pentanamine, and n-hexylmethylamine (2.67%).

Similarly, GC-MS analysis of *P. capillacea* extract (Table S3) showed N-hexadecanoic acid (palmitic acid) as the major constituent (39.31%), followed by 2,3-dimethoxyamphetamine, 4,4’-bis[4-methyl-2-pyrimidylsulfamido]terephthalanilide (9.44%), and a group of adrenergic agents including phenylephrine and metaraminol (7.21%).

### Molecular docking

**Fig. 7:**
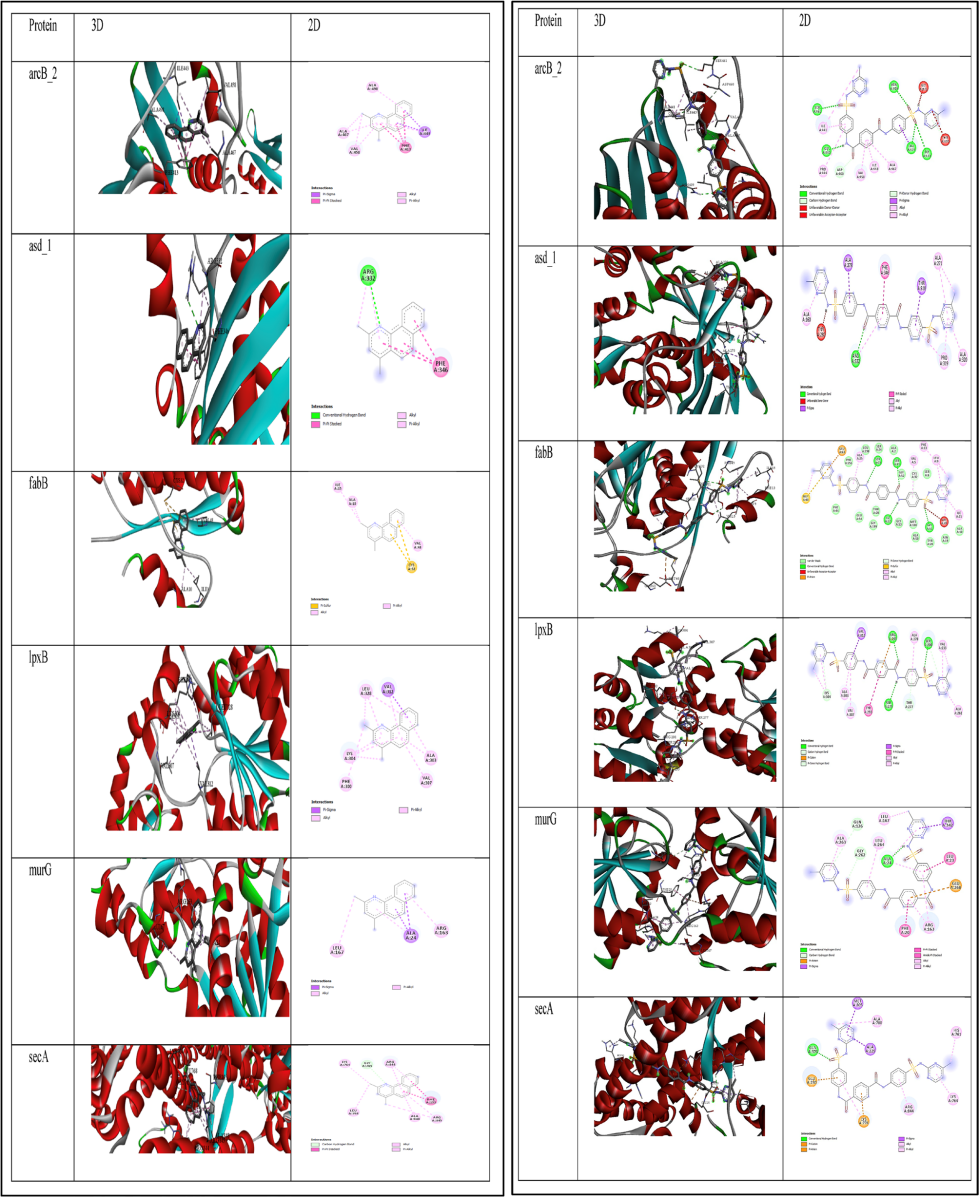
3D and 2D interactions for Benzo[h]quinoline_2_4_dimethyl_ (left panel) and 4,4'-Bis[4-methyl-2-pyrimidylsulfamido]terephthalanilide (right panel) with each protein

Docking results Binding affinity and ∆G (kcal/mol) for each compound of *Ulva Lactuca* extract with tested Proteins from *k. pneumoniae* is represented in (Table 2). However, the molecular docking results—expressed as binding affinities (∆G, kcal/mol)—for each identified compound from the *P. capillacea* extract against the tested *K. pneumoniae* protein targets are presented in (Table 3). Indicating that Benzo[h]quinoline-2,4-dimethyl and 4,4’-Bis[4-methyl-2-pyrimidylsulfamido]terephthalanilide are two compounds of the highest binding affinity to the six proteins of *k. pneumoniae.* The visualization of the representations of how Benzo[h]quinoline-2,4-dimethyl and 4,4’-Bis[4-methyl-2-pyrimidylsulfamido]terephthalanilide interact with each of the six protein targets is provided in (Fig. 7). The 3D images offer a spatial understanding of how the compound fits into the protein’s binding pocket, while the 2D diagrams illustrate the specific molecular interactions involved. These visualizations are crucial for understanding the binding mode and can guide future modifications to enhance the compound’s efficacy or specificity.

**Table 2:** Binding affinity and ΔG (kcal/mol) for each compound of *U. Lactuca* with tested Proteins from (*k. pneumoniae*)

Compound	arcB_2	asd_1	fabB	lpxB	murG	secA
1_2_Benzenediol_4_[2_(methylamino)ethyl]_	−6.1	−4.6	−6	−5.2	−6.3	−5.9
2_Aminononadecane	−6	−5	−6	−5.1	−6.2	−5.7
2_Amino_1_(o_hydroxyphenyl)propane	−5.6	−4.5	−5.5	−4.2	−4.4	−4.8
2_Butanamine_3_methyl	−4.1	−4.3	−4	−3.5	−4.1	−3.6
2_Pentanamine	−4.1	−3.2	−4	−3.4	−4.1	−3.6
3_3_Dimethyl_4_methylamino_butan_2_one	−4.5	−3.4	−4.6	−4.2	−4.7	−4.3
3_Ethoxyamphetamine	−6.6	−4.9	−6.1	−5.2	−6.4	−5.7
3_Methoxyamphetamine	−6.3	−4.9	−5.8	−5.1	−6.1	−5.5
Acetamide_2_fluoro	−3.4	−3.5	−3.5	−3.1	−3.6	−3.5
Allantoic_acid	−6.2	−4.9	−6.3	−5.2	−5.9	−6.1
Benzeneethanamine_4_fluoro__beta__3_dihydroxy_N_methyl_	−6.3	−4.9	−6.6	−5.4	−6.1	−6
Benzeneethanamine_N_methyl_	−6	−4.2	−5.7	−4.8	−5.3	−5
Benzo[h]quinoline_2_4_dimethyl_	**−10**	**−6.9**	**−8.8**	**−7.7**	**−6.6**	**−8.2**
dl_Phenylephrine	−6.1	−5	−6.3	−5	−6	−5.8
Ethanol_2_bromo_	−2.9	−3.1	−3	−2.6	−3	−2.9
Guanidinosuccinimide	−5.9	−4.9	−5.9	−5.1	−5.1	−5.4
L_Alanine_4_nitroanilide	−7	−5.1	−7.1	−5.7	−5.5	−6.3
Metanephrine	−5.9	−4.7	−6.1	−5.5	−6.2	−6.2
Methylpent_4_enylamine	−4	−3	−4.1	−3.4	−4.1	−3.6
Norephedrine	−6	−5.2	−6.1	−5.1	−6.6	−5.5
Norpseudoephedrine	−6.2	−4.7	−6.2	−5.5	−6	−5.7
n_Hexadecanoic_acid	−6.3	−4.7	−6.1	−4.9	−4.3	−5
n_Hexylmethylamine	−4.3	−3.1	−4.4	−3.6	−4.3	−3.7
N_Methyl_2_phenyl_1_propylamine	−6.4	−4.5	−6.2	−5.2	−5.8	−5.3
N_Methyl_3_(2_methylphenoxy)_3_phenylpropan_1_amine	−8.5	−5.8	−7.9	−6.2	−6.2	−7.3
Pentadecanoic_acid	−6.1	−4	−5.9	−4.6	−3.8	−5
Phenylephrine	−5.9	−4.9	−6.3	−5	−6	−5.7
Propanamide	−3.6	−3.8	−3.7	−3.2	−3.8	−3.4
sec_butyl_N_N_P_trimethylphosphonamidate	−4.8	−3.8	−5	−4.1	−3.9	−4.7
Tenamfetamine	−6.5	−5.2	−6.2	−5.4	−5.4	−6.1
Tetradecanoic_acid	−6.1	−4	−6.2	−5	−5.3	−5.5

**Table 3:** Binding affinity and ∆G (kcal/mol) for each compound of *P. capillacea *with tested Proteins from (*k. pneumoniae*)

S.N	Compound Name	arcB	asd_1	fabB	lpxB	murG	secA
1	[1,3]-Oxazino[5,6-c]quinoline,3-(3,4-methylenedioxybenzyl)−5-trifluoromethyl-3,4(2H)-dihydro-7-methoxy	−10.7	−8.0	−9.8	−8.7	−7.8	−9.5
2	2-Amino-N,Ndimethylethanesulfonamide	−4.5	−3.3	4.2	−3.9	−4.1	−4.2
3	2,3-Dimethoxyamphetamine	−6	−4.6	−6.1	−5.0	−4.9	−5.8
4	4,4'-Bis[4-methyl-2-pyrimidylsulfamido]terephthalanilide	−11.8	−8.8	−9.8	−9.6	−9.7	−10.6
5	n-Hexadecanoic acid	−6.4	−5.0	−6.8	−5.1	−5.3	−5.0
6	Ethylamine, 2-(adamantan-1-yl)−1-methyl-	−6.5	−5.1	−6.6	−5.4	−5.7	−6.1
7	4-Fluoroamphetamine	−6.2	−4.7	−6.0	−5.1	−5.5	−5.6
8	Metanephrine	−6	−4.9	−6.1	−5.5	−5.6	−6.3
9	Norephedrine, (.+/-.)-	−6	5.2	−5.8	−5.1	−6.7	−5.6
10	Benzeneethanamine, N-methyl-	−6.1	−4.5	−5.9	−4.9	−5.9	5.0
11	Benzeneethanamine, 4-fluoro-.beta.,3-dihydroxy-N-methyl-	−6.1	−4.9	−6.6	−5.5	−5.6	−6.1
12	Phenylephrine	−6.2	−5.0	−6.3	−5.1	−6.0	−5.8
13	Metaraminol	−6.2	−5.0	−6.4	−5.0	−5.8	−6.0
14	Acetamide, 2-fluoro-	−3.4	−3.5	−3.5	−3.2	−3.6	−3.6
15	Thiophene-3-ol, tetrahydro-, 1,1-dioxide	−4.6	−4.2	−4.5	−4.0	−4.7	−4.6
16	2-Butanamine, 3-methyl-	−4.1	−4.3	−4.0	−3.5	−4.1	−3.6
17	Methylpent-4-enylamine	−4.3	−4.0	−4.1	−3.6	−4.1	−3.7
18	Cyclobutanol	−4.2	−3.7	−3.7	−3.3	−3.8	−3.6
19	3,3-Dimethyl-4-methylamino-butan-2-one	−4.5	−4.5	−4.6	−4.0	−3.8	−4.3
20	Actinobolin	−6	−5.2	−6.6	−5.7	−6.2	−7.4
21	N-Desmethyltapentadol	−7.6	−4.9	−6.7	−5.8	−5.4	−6.2
22	Phenethylamine, p,.alpha.-dimethyl	−6.6	−4.7	−6.6	−5.3	−5.2	−5.8
23	Octodrine	−5.5	−3.6	−5.0	−4.4	−5.0	−4.6

The interactions of Benzo[h]quinoline-2,4-dimethyl with ArcB2 (Table S4) are predominantly hydrophobic, including pi-pi stacking with PHE413 and multiple pi-alkyl interactions. This suggests that the compound binds in a hydrophobic pocket of ArcB_2, which could potentially disrupt its function in the two-component signal transduction system of *K. pneumoniae*. However, A hydrogen bond with ARG332 is observed, along with pi-pi stacking with PHE346. This combination of hydrophobic and hydrogen bonding suggests a more specific interaction with ASD1 (Table S5), which could interfere with its role in amino acid biosynthesis. The interactions of Benzo[h]quinoline-2,4-dimethyl with FabB (Table S6) include pi-sulfur interactions with CYS32 and several hydrophobic interactions. This binding mode could potentially inhibit FabB’s function in fatty acid biosynthesis, a crucial process for bacterial cell membrane formation. The interactions of Benzo[h]quinoline-2,4-dimethyl with LpxB Multiple hydrophobic interactions are observed, particularly pi-alkyl interactions with various residues. This extensive network of hydrophobic interactions suggests strong binding to LpxB (Table S7), potentially disrupting lipid biosynthesis, which is essential for the outer membrane of gram-negative bacteria. The interactions of Benzo[h]quinoline-2,4-dimethyl with MurG (Table S8) is primarily hydrophobic, including pi-sigma interactions with ALA24. The binding to MurG could interfere with peptidoglycan biosynthesis, potentially weakening the bacterial cell wall.

In the interactions of Benzo[h]quinoline-2,4-dimethyl with SecA (Table S9), A carbon hydrogen bond with GLY765 is observed, along with pi-pi stacking with PHE193 and multiple hydrophobic interactions. This diverse interaction profile with SecA could disrupt protein translocation, a vital process for bacterial survival.

For 4,4’-Bis[4-methyl-2-pyrimidylsulfamido]terephthalanilide interaction with ASD1(Table S10), A carbon hydrogen bond with ARG332 is observed, along with pi-pi stacking with PHE346 and multiple hydrophobic interactions. This diverse interaction profile with ASD1 could disrupt protein translocation, a vital process for bacterial survival. The interaction with FabB (Table S11) include pi-sulfur interactions with MET60 and several hydrophobic interactions. This binding mode could potentially inhibit FabB’s function in fatty acid biosynthesis, a crucial process for bacterial cell membrane formation. The interactions of 4,4’-Bis[4-methyl-2-pyrimidylsulfamido]terephthalanilide with LpxB (Table S12), Multiple hydrophobic interactions are observed, particularly pi-alkyl interactions with various residues. This extensive network of hydrophobic interactions suggests strong binding to LpxB, potentially disrupting lipid biosynthesis, which is essential for the outer membrane of gram-negative bacteria. The interactions of 4,4’-Bis[4-methyl-2-pyrimidylsulfamido]terephthalanilide with MurG (Table S13) is primarily hydrophobic, including pi-sigma interactions with THR342. The binding to MurG could interfere with peptidoglycan biosynthesis, potentially weakening the bacterial cell wall. In the interactions of 4,4’-Bis[4-methyl-2-pyrimidylsulfamido]terephthalanilide with SecA (Table S14 & S15), Multiple hydrophobic interactions are observed, particularly pi-alkyl interactions with various residues. This extensive network of hydrophobic interactions suggests strong binding to SecA and arcB_2, potentially disrupting lipid biosynthesis, which is essential for the outer membrane of gram-negative bacteria.

### In silico toxicity test

**Fig. 8:**
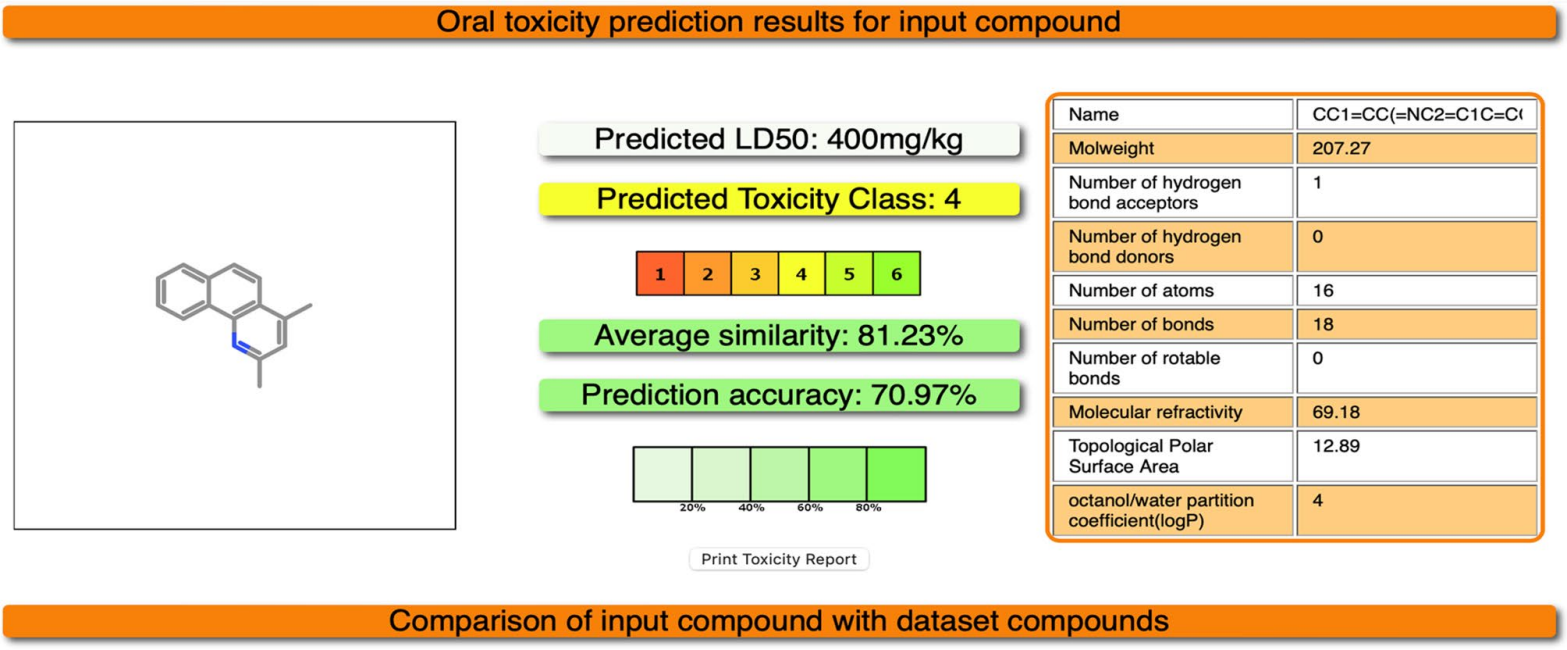
In silico toxicity of Benzo[h]quinoline_2_4_dimethyl_ using https://tox.charite.de/protox3/index.php?site=home

**Fig. 9:**
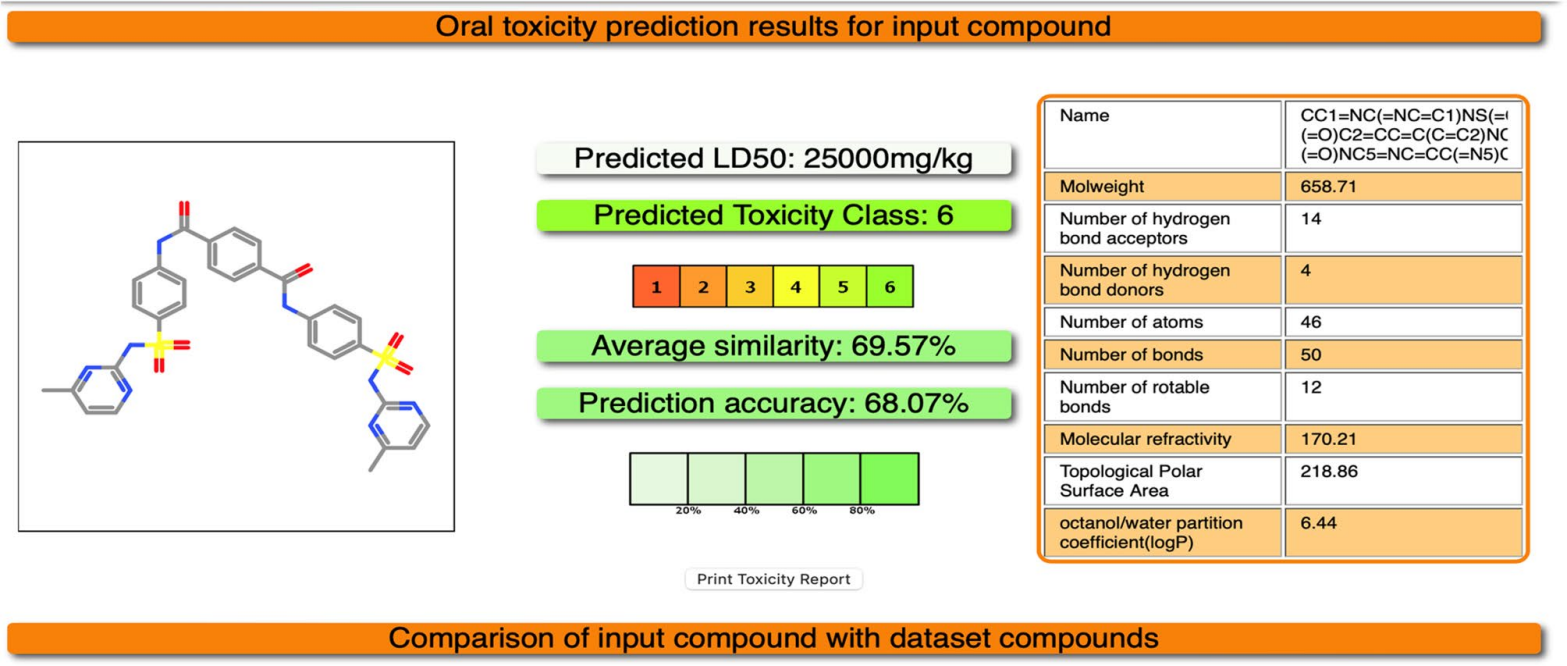
In silico toxicity of 4,4’-Bis[4-methyl-2-pyrimidylsulfamido]terephthalanilide using https://tox.charite.de/protox3/index.php?site=home

Testing toxicity of the most effective compounds against *k. pneumoniae* using protox3 website indicate that the toxicity of Benzo[h]quinoline_2_4_dimethyl_ is of class 4 with average similarity of 81.23% and prediction accuracy of 70.97% depending on the predicted LD50 400 mg/kg (Fig. 8) however, the toxicity of 4,4’-Bis[4-methyl-2-pyrimidylsulfamido]terephthalanilide is of class 6, which is the least toxicity class with a similarity average of 69.57% and a predicted accuracy 68.07% (Fig. 9).

## Discussion

The primary cause for concern these days is the antimicrobial resistance of bacterial strains to the majority of common antibiotics [68]. Because of the rise of antibiotic resistance, antibiotic alternatives are urgently needed [1]. Globally, seaweeds are extensively screened to separate drugs or bioactive compounds [69].

Al-Saif SSA et al. and Wijesekara et al. [15, 16] demonstrated that chemicals originating from marine algae have a wide range of biological functions. Two marine algae’s antibacterial activity has been screened, and their extracts have been assessed as a potential substitute for widely used antibiotics [70].

Accordingly, the present study was focused to screen two seaweeds viz., *U. lactuca*,* P. capillacea* using two distinct solvents to assess the antibacterial activity potential. Seaweed demonstrated distinct antibacterial properties against a human bacterial pathogen in a range of solvents viz., *K. pneumoniae.*

The present study coincides with [71], that used ethanolic and methanolic extracts of seaweeds exhibited a higher zone of inhibition against both gram–positive such as *Staphylococcus aureus* and gram–negative bacteria such as *Pseudomonas aeruginosa* and *Klebsiella pneumoniae*. In the present study ethanolic extract of *Pterocladiella capillacea* displayed highest inhibitory activity against *K. pneumonia.* [71–74] found that the antimicrobial activity of *Ulva* extracts showed activity against *Klebsiella pneumoniae*,* Staphylococcus*, and *Pseudomonas aeruginosa.*

Osman, Vimala and Poonghuzhali [74, 75] reported that maximum activities were recorded by ethanolic extract showed maximum activity against *K. pneumoniae* (17 mm). Similarly, in this study, ethanolic extract showed maximum activity against *K. pneumoniae* (27 mm). In addition, a synergistic effect was observed for the combination of algae or cyanobacteria with algae [44, 46, 47]. found that synergistic antibacterial effects of algal methanolic extract alone and in combination with different antibiotics where, using a well diffusion assay against multidrug- resistant *K. pneumoniae*, recorded a highest inhibition zone diameter of (*Ulva* + Gentamicin) 25 mm. Consequently, synergistic antibacterial effects of our study, recorded a highest inhibition zone diameter of (*U. lactuca* + *Pterocladiella capillacea.* + Nitrofurantoin) 36 mm against *K. pneumoniae*, which mean increasing the inhibition zone by 100%.

This study employed molecular docking techniques to examine how various compounds interact with six key proteins from *K. pneumoniae*: ArcB, Asd, FabB, LpxB, MurG, and SecA. These proteins, essential for different bacterial functions, represent potential targets for new antibacterial drugs. The research aimed to identify compounds that bind strongly to these proteins, potentially leading to new treatments against *K. pneumoniae*.

Benzo[h]quinoline-2,4-dimethyl and 4,4’-Bis[4-methyl-2-pyrimidylsulfamido]terephthalanilide emerged as the standout compounds, consistently demonstrating the highest binding affinity across all six protein targets. They showed particularly strong interactions with ArcB, FabB, and SecA, with binding energies of −10, −8.8, and − 8.2 kcal/mol respectively for Benzo[h]quinoline-2,4-dimethyl and − 11.8, −9.8 and − 10.6 kcal/mol respectively for 4,4’-Bis[4-methyl-2-pyrimidylsulfamido]terephthalanilide. Such high binding affinities suggest these compounds could effectively inhibit or alter the function of these proteins, potentially disrupting critical bacterial processes.

The interactions between these compounds and the target proteins were primarily hydrophobic, including pi-pi stacking, pi-alkyl, and alkyl interactions. These non-covalent bonds are crucial for stabilizing the ligand-protein complex and significantly contribute to the overall binding strength. The prevalence of hydrophobic interactions suggests the compound may effectively penetrate the bacterial cell membrane, an important factor for potential antibacterial agents.

In some instances, such as with ASD1 and SecA, hydrogen bonding was also observed. These hydrogen bonds can enhance binding specificity and may be vital for the compound’s biological activity. The combination of hydrophobic interactions and hydrogen bonds indicates that these compounds might offer a good balance between membrane permeability and target specificity.

The compound’s strong interactions with multiple bacterial targets suggest it could potentially act as a multi-target inhibitor. This characteristic is highly desirable in antibacterial drug development, as it may reduce the likelihood of rapid resistance development and potentially result in more effective treatments against *K. pneumoniae* infections.

In each case, the predominance of hydrophobic interactions suggests that both Benzo[h]quinoline-2,4-dimethyl and 4,4’-Bis[4-methyl-2-pyrimidylsulfamido]terephthalanilide bind strongly in hydrophobic pockets of these proteins. The occasional hydrogen bonds or other specific interactions may contribute to binding specificity. The consistent strong binding across all six targets indicates that this compound has the potential to disrupt multiple essential bacterial processes simultaneously, which could make it a promising lead for antibacterial drug development against *K. pneumoniae*.

## Conclusion

Antibiotic resistance against a wide spectrum of pathogens and bacteria can result from the irrational use of antibiotics. Because of the development of antibiotic resistance, antibiotic alternatives are urgently required. The results we obtained in this study can be summarized in the following points: Two species of marine algae were collected from Rocky Bay of Abu Qir, Alexandria coast, Egypt. A red algae, *Pterocladiella capillacea* as well as one green alga, *Ulva lactuca*, different concentrations (500, 250, 125, 62.5, and 31.25 mg/ml) of the algal extracts were prepared using absolute ethanol and methanol. Application of different algal extracts on bacterial isolates, the ethanolic extract recorded the highest results was *P. capillacea*, followed by *U. lactuca* against *Klebsiella pneumoniae*. After synergetic inhibition effect, the most effective mixture on *K. pneumoniae* was the mixture of (*U. lactuca + P. capillacea.* + Nitrofurantoin), which increased the inhibition zone by 100%. GC-MS analysis of the extracts *P. capillacea* and *U. lactuca* revealed several components, indicating antibacterial activity. After screening of different bioactive compounds of algal extracts, the most effective compounds were: Benzo[h] quinoline_2_4_dimethyl_ and 4, 4’-Bis [4-methyl-2-pyrimidylsulfamido] terephthalanilide. These two substances are interesting candidates for the development of novel antimicrobial drugs due to their efficacy against strains of bacteria that are resistant to antibiotics. that was confirmed by molecular docking of these compounds with the most targeted proteins of *K. pneumoniae* by antibiotics.

## Supplementary Information

Supplementary Material 1.

## Data Availability

The identified strain was submitted in the gene bank under the accession number PP439638.1. https://www.ncbi.nlm.nih.gov/nuccore/PP439638.1/.
