# Supplementary material for: Synergetic effects of Ulva lactuca and Pterocladiella capillacea on the multidrug-resistant Klebsiella pneumoniae
Source: BMC Microbiol. 2025 Jun 26;25:370. doi: 10.1186/s12866-025-04102-4 (PMC12199523; doi:10.1186/s12866-025-04102-4)
Supplement: Supplementary file 1 — Supplementary Material 1. [file 12866_2025_4102_MOESM1_ESM.docx]

**Table S1.** Morphological and some biochemical characteristics of ***K. pneumoniae*** isolate.

| Test | *K. pneumoniae* | |
| --- | --- | --- |
|  | MacConkey agar | Blood Agar |
| Colony shape | Circular | Circular |
| Colony size | 2-3 mm | 2-3 mm |
| Colony Elevation | Convex | Dome-shaped |
| Colony Surface | Mucoid | Mucoid |
| Colony Color | Pink – Red | Greyish white |
| Gram reaction | Negative | |
| bacterial Shape | rods | |
| Coagulase test | Negative | |
| Hemolysis on blood agar |  | γ-Hemolysis  (Non-hemolytic) |

**
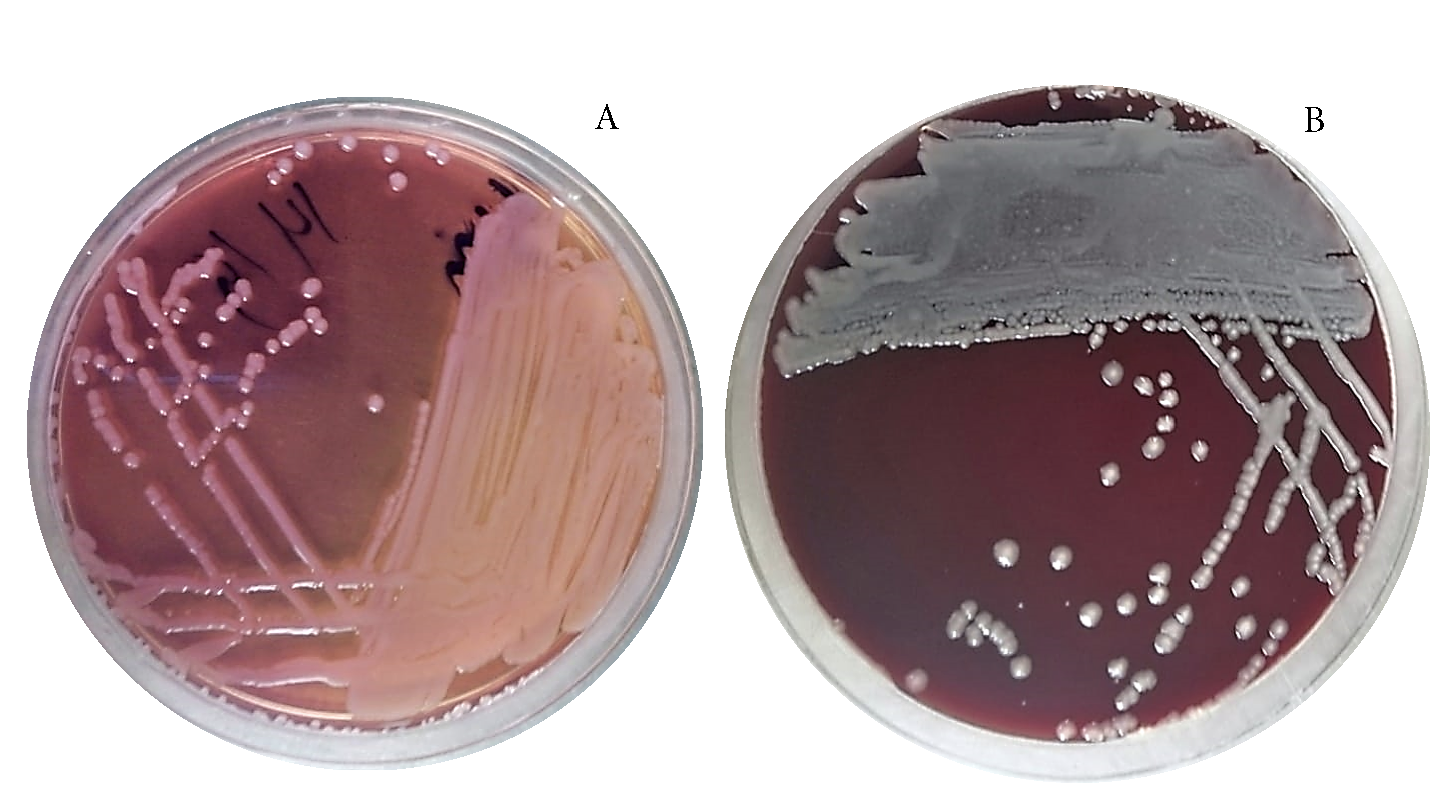
**

**Figure S1.** Morphology of *K. pneumoniae* A) On Blood Agar as Dome-shaped and greyish white and B) On MacConkey agar as pink colored.


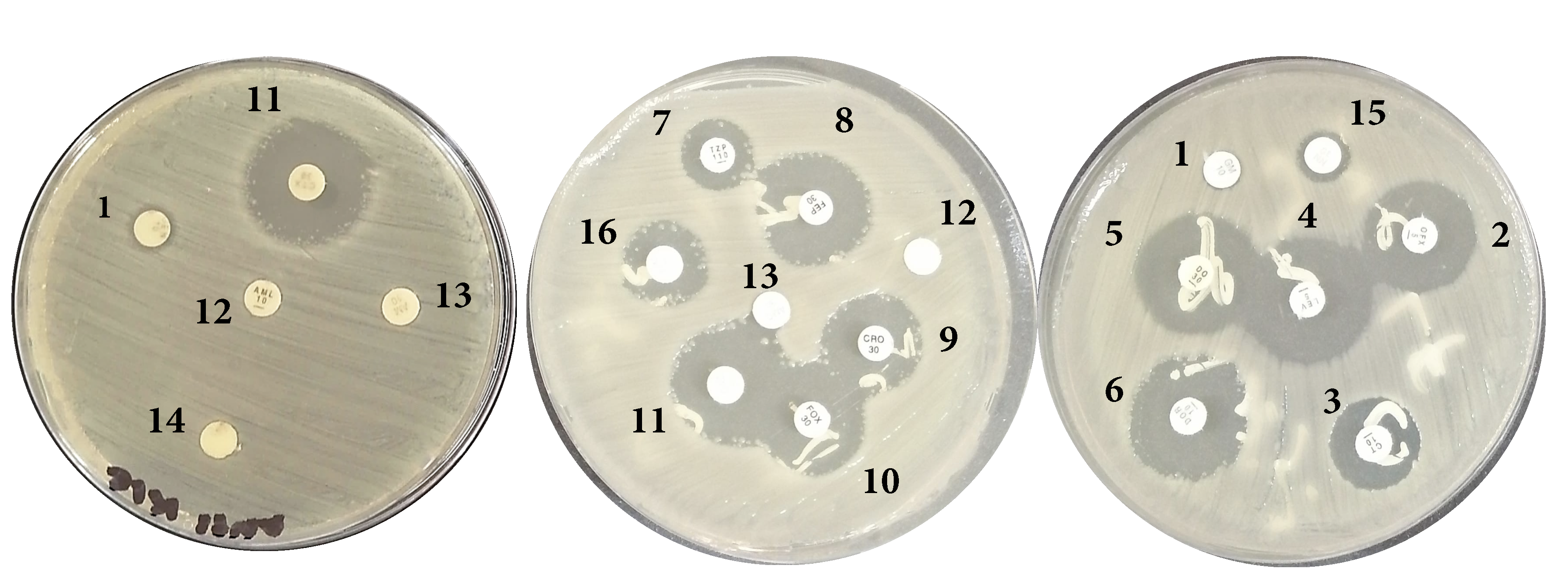


Figure S2: Sixteen antibiotics were used against K. pneumoniae; 1- Gentamicin (GM), 2- Ofloxacin (OFX), 3- Colistin (CT), 4- Levofloxacin (LEV),5- Doxycycline (DOR) 7- Piperacillin/Tazobactam (TZP), 8- Cefepim (FEP), 9- Ceftriaxone (CRO), 10- Cefoxitin (FOX), 11- Cefotaxime (CTX), 12- Amoxicillin (AML), 13- Ampicillin (AM), 14- Ampicillin/Sulbactam (SAM), 15- Tobramycin (TOB) and 16- Ceftazidime (CAZ).

**Table S2.** Gas chromatography (GC) analysis of *Ulva lactuca* extract.

| **S.N** | **RT(min)** | **Compound Name** | **Molecular Formula** | **MW** | **Peak area** |
| --- | --- | --- | --- | --- | --- |
| **1** | 14.724 | Allantoic acid | C₄H₈N₄O₄ | 176.1307 | 2.83 |
|  |  | Tetradecanoic acid | C₁₄H₂₈O₂ | 228.37 |  |
|  |  | Pentadecanoic acid | C₁₅H₃₀O₂ | 242.3975 |  |
| **2** | 16.327 | L-Alanine-4-nitroanilide | C_9_H_11_N_3_O_3_ | 209.2 | 1.74 |
|  |  | Tenamfetamine | C10H13NO2 | 179.2157 |  |
|  |  | Phenylephrine | C_9_H_13_NO_2_ | 167.205 |  |
| **3** | 17.16 | dl-Phenylephrine |  |  | 1.7 |
|  |  | 1,2-Benzenediol, 4-[2-(methylamino)ethyl]- | C_9_H_13_NO_2_ | 167.21 |  |
|  |  | Norephedrine, (.+/-.)- | C_9_H_13_NO | 151.2056 |  |
| **4** | 18.746 | n-Hexadecanoic acid | C₁₆H₃₂O₂ | 256.4241 | 73.57 |
| **5** | 19.066 | l-Guanidinosuccinimide | C_5_H_7_N_3_O_2_ | 141.13 | 0.91 |
|  |  | N-Methyl-2-phenyl-1-propylamine |  |  |  |
|  |  | 3,3-Dimethyl-4-methylamino-butan-2-one | C_7_H_15_NO | 129.2 |  |
| **6** | 19.253 | Acetamide, 2-fluoro- | C_2_H_4_FNO | 77.0577 | 0.43 |
|  |  | Metanephrine | C₁₀H₁₅NO₃ | 197.231 |  |
|  |  | 3-Ethoxyamphetamine | C_11_H_17_NO | 179.26 |  |
| **7** | 21.485 | sec-butyl N,N,P-trimethylphosphonamidate |  |  | 4.77 |
|  |  | 2-Amino-1-(o-hydroxyphenyl)propane |  |  |  |
|  |  | Ethanol, 2-bromo- | C2H5BrO | 124.964 |  |
| **8** | 21.905 | Benzeneethanamine, N-methyl- | C_9_H_13_N | 135.2062 | 1.92 |
|  |  | N-Methyl-3-(2-methylphenoxy)-3-phenylpropan-1-amine | C_34_H_41_Cl_2_F_3_N_2_O_2_ | 637.6 |  |
| **9** | 21.987 | 3-Methoxyamphetamine | C_10_H_15_NO | 165.23 | 4.38 |
|  |  | Norpseudoephedrine | C_9_H_13_NO | 151.2056 |  |
| **10** | 22.33 | Propanamide | C_3_H_7_NO | 73.095 | 1.54 |
|  |  | Methylpent-4-enylamine | C_6_H_13_N | 99.17 |  |
| **11** | 22.447 | 2-Aminononadecane | C_19_H_41_N | 283.535 | 1.13 |
| **12** | 27.984 | 2-Butanamine, 3-methyl- | C5H13N | 87.1634 | 1.27 |
| **13** | 28.649 | Benzeneethanamine, 4-fluoro-.beta.,3-dihydroxy-N-methyl- | C_9_H_12_FNO_2_ | 185.2 | 1.14 |
| **14** | 45.884 | Benzo[h]quinoline, 2,4-dimethyl- | C15H13N | 207.2704 | 2.67 |
|  |  | 2-Pentanamine | C5H13N | 87.1634 |  |
|  |  | n-Hexylmethylamine | C_7_H_17_N | 115.22 |  |

**Table S3.** Gas chromatography (GC) analysis of *Pterocladiella capillacea* extract.

| **S.N** | **RT(min)** | **Compound Name** | **Molecular Formula** | **MW** | **Peak area** |
| --- | --- | --- | --- | --- | --- |
| **1** | 14.712 | [1,3]-Oxazino[5,6-c]quinoline,3-(3,4-methylenedioxybenzyl)-5-trifluoromethyl-3,4(2H)-dihydro-7-methoxy |  |  | 6 |
|  |  | Benzamide, 3-methoxy-N-(2-butyl)-N -tetradecyl- |  |  |  |
|  |  | l-Alanine, N-octanoyl-, tetradecylester |  |  |  |
| **2** | 14.835 | 2-Amino-N,N-dimethylethanesulfonamide | C_4_H_12_N_2_O_2_S | 152.22 | 6.49 |
|  |  |  |  |  |  |
|  |  | Benzamide, 3-methoxy-N-(2-butyl)-N-tetradecyl- | C_2_H_4_FNO | 77.0577 |  |
| **3** | 15.004 | 2,3-Dimethoxyamphetamine | C_11_H_17_NO_2_ | 195.26 | 9.44 |
|  |  | 4,4'-Bis[4-methyl-2-pyrimidylsulfamido]terephthalanilide |  |  |  |
|  |  |  |  |  |  |
| **4** | 18.67 | n-Hexadecanoic acid | C₁₆H₃₂O₂ | 256.424 | 39.31 |
| **5** | 20.034 | Ethylamine, 2-(adamantan-1-yl)-1-methyl- | C_13_H_23_N | 193.33 | 2.98 |
|  |  | 4-Fluoroamphetamine | C_9_H_12_FN | 153.2 |  |
|  |  | Metanephrine | C₁₀H₁₅NO₃ | 197.231 |  |
| **6** | 21.479 | Norephedrine, (.+/-.)- | C_9_H_13_NO | 151.206 | 4.08 |
|  |  | Benzeneethanamine, N-methyl- | C_9_H_13_N | 135.206 |  |
|  |  | Benzeneethanamine, 4-fluoro-.beta.,3-dihydroxy-N-methyl- | C_9_H_12_FNO_2_ | 185.2 |  |
| **7** | 21.893 | Phenylephrine | C_9_H_13_NO_2_ | 167.205 | 7.21 |
|  |  | Metaraminol | C_9_H_13_NO_2_ | 167.208 |  |
|  |  |  |  |  |  |
| **8** | 21.963 | Acetamide, 2-fluoro- | C_2_H_4_FNO | 77.0577 | 5.1 |
|  |  | Thiophene-3-ol, tetrahydro-, 1,1-dioxide |  |  |  |
|  |  | 2-Butanamine, 3-methyl- | C_5_H_13_N | 87.1634 |  |
| **9** | 22.319 | Acetamide, 2-fluoro- | C_2_H_4_FNO | 77.0577 | 6.14 |
|  |  | Methylpent-4-enylamine | C_6_H_13_N | 99.17 |  |
|  |  |  |  |  |  |
| **10** | 23.473 | Norephedrine, (.+/-.)- | C_9_H_13_NO | 151.206 | 4.31 |
|  |  | Cyclobutanol | C₄H₈O | 72.1062 |  |
|  |  | Benzeneethanamine, 4-fluoro-.beta.,3-dihydroxy-N-methyl- | C_9_H_12_FNO_2_ | 185.2 |  |
| **11** | 28.002 | Benzeneethanamine, N-methyl- | C_9_H_13_N | 135.206 | 3.95 |
|  |  | 3,3-Dimethyl-4-methylamino-butan-2-one | C_7_H_15_NO | 129.2 |  |
|  |  | Actinobolin | C_13_H_20_N_2_O_6_ | 300.308 |  |
| **12** | 41.14 | N-Desmethyltapentadol | C_13_H_21_NO | 207.31 | 5 |
|  |  | Phenethylamine, p,.alpha.-dimethyl | C_10_H_15_N | 149.233 |  |
|  |  | Octodrine | C_8_H_19_N | 129.24 |  |

**Table S4:** Best compound with ArcB2

| Interaction | Distance | Category | Type |
| --- | --- | --- | --- |
| A:ILE443:CD1 - :UNL1 | 3.75927 | Hydrophobic | Pi-Sigma |
| A:ILE443:CD1 - :UNL1 | 3.72192 | Hydrophobic | Pi-Sigma |
| A:PHE413 - :UNL1 | 4.08199 | Hydrophobic | Pi-Pi Stacked |
| A:PHE413 - :UNL1 | 3.71483 | Hydrophobic | Pi-Pi Stacked |
| A:PHE413 - :UNL1 | 4.20699 | Hydrophobic | Pi-Pi Stacked |
| A:ALA467 - :UNL1:C | 3.79585 | Hydrophobic | Alkyl |
| :UNL1:C - A:VAL458 | 3.98329 | Hydrophobic | Alkyl |
| :UNL1 - A:ILE443 | 5.47837 | Hydrophobic | Pi-Alkyl |
| :UNL1 - A:VAL458 | 4.23594 | Hydrophobic | Pi-Alkyl |
| :UNL1 - A:ALA467 | 4.24928 | Hydrophobic | Pi-Alkyl |
| :UNL1 - A:ALA498 | 4.58133 | Hydrophobic | Pi-Alkyl |

**Table S5:** Best ligand with ASD1

| Interaction | distance | Category | Type |
| --- | --- | --- | --- |
| A:ARG332:HH12 - :UNL1:N | 2.74808 | Hydrogen Bond | Conventional Hydrogen Bond |
| A:PHE346 - :UNL1 | 4.68989 | Hydrophobic | Pi-Pi Stacked |
| A:PHE346 - :UNL1 | 3.91819 | Hydrophobic | Pi-Pi Stacked |
| A:PHE346 - :UNL1 | 3.72183 | Hydrophobic | Pi-Pi Stacked |
| :UNL1:C - A:ARG332 | 3.74574 | Hydrophobic | Alkyl |
| :UNL1 - A:ARG332 | 5.19742 | Hydrophobic | Pi-Alkyl |

**Table S6:** Best ligand with FabB

| Interaction | Distance | Category | Type |
| --- | --- | --- | --- |
| A:CYS32:SG - :UNL1 | 5.08691 | Other | Pi-Sulfur |
| A:CYS32:SG - :UNL1 | 5.39831 | Other | Pi-Sulfur |
| A:ALA10 - :UNL1:C | 3.4335 | Hydrophobic | Alkyl |
| :UNL1:C - A:ILE15 | 4.08631 | Hydrophobic | Alkyl |
| :UNL1 - A:VAL48 | 5.22017 | Hydrophobic | Pi-Alkyl |

**Table S7:** Interaction between IpxB and best ligand

| Interaction | distance | Category | Type |
| --- | --- | --- | --- |
| A:VAL312:CG2 - :UNL1 | 3.71136 | Hydrophobic | Pi-Sigma |
| :UNL1:C - A:LYS304 | 4.28728 | Hydrophobic | Alkyl |
| :UNL1:C - A:LEU328 | 4.90196 | Hydrophobic | Alkyl |
| A:PHE300 - :UNL1:C | 5.33745 | Hydrophobic | Pi-Alkyl |
| :UNL1 - A:LYS304 | 4.40986 | Hydrophobic | Pi-Alkyl |
| :UNL1 - A:LEU328 | 5.47208 | Hydrophobic | Pi-Alkyl |
| :UNL1 - A:ALA303 | 4.45832 | Hydrophobic | Pi-Alkyl |
| :UNL1 - A:LYS304 | 5.36693 | Hydrophobic | Pi-Alkyl |
| :UNL1 - A:VAL307 | 4.65441 | Hydrophobic | Pi-Alkyl |
| :UNL1 - A:VAL312 | 4.89913 | Hydrophobic | Pi-Alkyl |
| :UNL1 - A:ALA303 | 4.86058 | Hydrophobic | Pi-Alkyl |
| :UNL1 - A:LEU328 | 4.79588 | Hydrophobic | Pi-Alkyl |

**Table S8:** Interaction between MurG and best ligand

| Interaction | Distance | Category | Type |
| --- | --- | --- | --- |
| A:ALA24:CB - :UNL1 | 3.69191 | Hydrophobic | Pi-Sigma |
| A:ALA24:CB - :UNL1 | 3.88821 | Hydrophobic | Pi-Sigma |
| :UNL1:C - A:LEU167 | 5.39583 | Hydrophobic | Alkyl |
| :UNL1 - A:ALA24 | 4.26243 | Hydrophobic | Pi-Alkyl |
| :UNL1 - A:ARG163 | 5.11849 | Hydrophobic | Pi-Alkyl |

**Table S9:** Interaction between SecA and best ligand

| Interaction | distance | Category | Type |
| --- | --- | --- | --- |
| A:GLY765:CA - :UNL1:N | 3.67987 | Hydrogen Bond | Carbon Hydrogen Bond |
| A:PHE193 - :UNL1 | 4.66315 | Hydrophobic | Pi-Pi Stacked |
| A:PHE193 - :UNL1 | 5.09989 | Hydrophobic | Pi-Pi Stacked |
| :UNL1:C - A:ARG845 | 4.87901 | Hydrophobic | Alkyl |
| :UNL1:C - A:LYS764 | 3.87972 | Hydrophobic | Alkyl |
| :UNL1:C - A:LEU768 | 5.37933 | Hydrophobic | Alkyl |
| :UNL1 - A:ARG844 | 4.6183 | Hydrophobic | Pi-Alkyl |
| :UNL1 - A:ARG845 | 5.39069 | Hydrophobic | Pi-Alkyl |
| :UNL1 - A:ALA848 | 5.18424 | Hydrophobic | Pi-Alkyl |
| :UNL1 - A:ARG844 | 3.87489 | Hydrophobic | Pi-Alkyl |

| **Table S10: Best compound with asd_1** | | | |
| --- | --- | --- | --- |
| Interaction | Distance | Category | Type |
| A:ARG332:HH11 - :UNL1:O | 2.53873 | Hydrogen Bond | Conventional Hydrogen Bond |
| A:ALA278:CB - :UNL1 | 3.50694 | Hydrophobic | Pi-Sigma |
| A:THR318:CG2 - :UNL1 | 3.53423 | Hydrophobic | Pi-Sigma |
| A:PHE346 - :UNL1 | 3.78855 | Hydrophobic | Pi-Pi Stacked |
| A:ALA271 - :UNL1:C | 3.55498 | Hydrophobic | Alkyl |
| :UNL1 - A:ARG332 | 5.48678 | Hydrophobic | Pi-Alkyl |
| :UNL1 - A:PRO319 | 5.45851 | Hydrophobic | Pi-Alkyl |
| :UNL1 - A:ALA271 | 4.91054 | Hydrophobic | Pi-Alkyl |
| :UNL1 - A:PRO319 | 3.90354 | Hydrophobic | Pi-Alkyl |
| :UNL1 - A:ALA320 | 4.87193 | Hydrophobic | Pi-Alkyl |
| :UNL1 - A:ALA160 | 4.59062 | Hydrophobic | Pi-Alkyl |

| **Table S11: Best compound with fabB_1** | | | |
| --- | --- | --- | --- |
| Interaction | Distance | Category | Type |
| A:SER21:HG - :UNL1:O | 2.60675 | Hydrogen Bond | Conventional Hydrogen Bond |
| A:GLY51:HN - :UNL1:O | 2.78152 | Hydrogen Bond | Conventional Hydrogen Bond |
| :UNL1:H - A:ILE22:O | 3.06008 | Hydrogen Bond | Conventional Hydrogen Bond |
| :UNL1:H - A:SER23:OG | 2.61203 | Hydrogen Bond | Conventional Hydrogen Bond |
| A:GLU63:OE2 - :UNL1 | 4.061 | Electrostatic | Pi-Anion |
| A:CYS32:SG - :UNL1 | 3.88542 | Hydrogen Bond | Pi-Donor Hydrogen Bond |
| A:MET60:SD - :UNL1 | 5.97075 | Other | Pi-Sulfur |
| :UNL1:C - A:LEU9 | 4.31464 | Hydrophobic | Alkyl |
| :UNL1:C - A:MET60 | 4.30195 | Hydrophobic | Alkyl |
| A:PHE13 - :UNL1:C | 5.00366 | Hydrophobic | Pi-Alkyl |
| A:PHE49 - :UNL1:C | 4.69367 | Hydrophobic | Pi-Alkyl |
| :UNL1 - A:VAL5 | 4.61662 | Hydrophobic | Pi-Alkyl |
| :UNL1 - A:LEU9 | 5.46821 | Hydrophobic | Pi-Alkyl |
| :UNL1 - A:ILE15 | 4.96184 | Hydrophobic | Pi-Alkyl |
| :UNL1 - A:ALA25 | 3.9215 | Hydrophobic | Pi-Alkyl |

| **Table S12: Best compound with lpxB** | | | |
| --- | --- | --- | --- |
| Interaction | Distance | Category | Type |
| A:SER200:HG - :UNL1:O | 2.64605 | Hydrogen Bond | Conventional Hydrogen Bond |
| A:ARG201:HH21 - :UNL1:O | 2.53819 | Hydrogen Bond | Conventional Hydrogen Bond |
| :UNL1:H - A:SER275:O | 2.35777 | Hydrogen Bond | Conventional Hydrogen Bond |
| A:SER200:CB - :UNL1:N | 3.72394 | Hydrogen Bond | Carbon Hydrogen Bond |
| A:LYS304:CA - :UNL1:N | 3.44168 | Hydrogen Bond | Carbon Hydrogen Bond |
| A:ARG201:NH1 - :UNL1 | 4.15274 | Hydrogen Bond;Electrostatic | Pi-Cation;Pi-Donor Hydrogen Bond |
| A:THR277:HN - :UNL1 | 2.91332 | Hydrogen Bond | Pi-Donor Hydrogen Bond |
| A:VAL312:CG1 - :UNL1 | 3.77055 | Hydrophobic | Pi-Sigma |
| A:VAL312:CG2 - :UNL1 | 3.77905 | Hydrophobic | Pi-Sigma |
| A:TYR293 - :UNL1 | 5.36352 | Hydrophobic | Pi-Pi Stacked |
| :UNL1:C - A:VAL233 | 4.12068 | Hydrophobic | Alkyl |
| :UNL1:C - A:LYS304 | 4.24337 | Hydrophobic | Alkyl |
| :UNL1 - A:ALA278 | 5.36011 | Hydrophobic | Pi-Alkyl |
| :UNL1 - A:VAL233 | 4.667 | Hydrophobic | Pi-Alkyl |
| :UNL1 - A:ALA261 | 4.28938 | Hydrophobic | Pi-Alkyl |
| :UNL1 - A:ALA303 | 4.51868 | Hydrophobic | Pi-Alkyl |
| :UNL1 - A:VAL307 | 5.22842 | Hydrophobic | Pi-Alkyl |
| :UNL1 - A:LYS304 | 5.17874 | Hydrophobic | Pi-Alkyl |

| **Table S13: Best compound with murG** | | | |
| --- | --- | --- | --- |
| Interaction | Distance | Category | Type |
| :UNL1:H - A:ALA24:O | 2.64377 | Hydrogen Bond | Conventional Hydrogen Bond |
| A:GLY262:CA - :UNL1:O | 3.51552 | Hydrogen Bond | Carbon Hydrogen Bond |
| :UNL1:C - A:GLN126:OE1 | 3.58234 | Hydrogen Bond | Carbon Hydrogen Bond |
| A:GLU268:OE1 - :UNL1 | 4.8204 | Electrostatic | Pi-Anion |
| A:THR342:CG2 - :UNL1 | 3.8025 | Hydrophobic | Pi-Sigma |
| A:PHE20 - :UNL1 | 3.73262 | Hydrophobic | Pi-Pi Stacked |
| A:LEU23:C,O;ALA24:N - :UNL1 | 5.16592 | Hydrophobic | Amide-Pi Stacked |
| :UNL1:C - A:LEU167 | 5.38066 | Hydrophobic | Alkyl |
| :UNL1 - A:ARG163 | 4.52911 | Hydrophobic | Pi-Alkyl |
| :UNL1 - A:ALA24 | 4.01917 | Hydrophobic | Pi-Alkyl |
| :UNL1 - A:ARG163 | 5.35741 | Hydrophobic | Pi-Alkyl |
| :UNL1 - A:ALA24 | 4.91313 | Hydrophobic | Pi-Alkyl |
| :UNL1 - A:LEU264 | 5.17654 | Hydrophobic | Pi-Alkyl |
| :UNL1 - A:ALA263 | 4.94089 | Hydrophobic | Pi-Alkyl |

| Table S14: Best compound with secA | | | |
| --- | --- | --- | --- |
| Interaction | Distance | Category | Type |
| A:GLN370:HE21 - :UNL1:O | 2.19051 | Hydrogen Bond | Conventional Hydrogen Bond |
| A:LYS776:NZ - :UNL1 | 3.82717 | Electrostatic | Pi-Cation |
| A:GLU230:OE1 - :UNL1 | 3.71324 | Electrostatic | Pi-Anion |
| A:ALA229:CB - :UNL1 | 3.68649 | Hydrophobic | Pi-Sigma |
| A:MET305:CE - :UNL1 | 3.97964 | Hydrophobic | Pi-Sigma |
| A:ALA780 - :UNL1:C | 4.42895 | Hydrophobic | Alkyl |
| A:HIS761 - :UNL1:C | 4.56365 | Hydrophobic | Pi-Alkyl |
| :UNL1 - A:ARG844 | 4.07309 | Hydrophobic | Pi-Alkyl |
| :UNL1 - A:LYS764 | 4.88234 | Hydrophobic | Pi-Alkyl |
| :UNL1 - A:LYS776 | 4.82252 | Hydrophobic | Pi-Alkyl |

| Table S15: Best compound with arcB_2 | | | |
| --- | --- | --- | --- |
| Interaction | Distance | Category | Type |
| A:ASN409:HD21 - :UNL1:O | 2.38763 | Hydrogen Bond | Conventional Hydrogen Bond |
| A:SER461:HG - :UNL1:O | 2.70489 | Hydrogen Bond | Conventional Hydrogen Bond |
| A:GLY473:HN - :UNL1:O | 2.67713 | Hydrogen Bond | Conventional Hydrogen Bond |
| A:LEU474:HN - :UNL1:O | 1.86223 | Hydrogen Bond | Conventional Hydrogen Bond |
| :UNL1:H - A:GLY442:O | 1.94369 | Hydrogen Bond | Conventional Hydrogen Bond |
| A:ASP460:CA - :UNL1:O | 3.72478 | Hydrogen Bond | Carbon Hydrogen Bond |
| A:THR470:HN - :UNL1 | 2.88587 | Hydrogen Bond | Pi-Donor Hydrogen Bond |
| A:LEU474:CD1 - :UNL1 | 3.80873 | Hydrophobic | Pi-Sigma |
| :UNL1:C - A:ILE441 | 4.91823 | Hydrophobic | Alkyl |
| :UNL1 - A:ILE443 | 5.06516 | Hydrophobic | Pi-Alkyl |
| :UNL1 - A:VAL458 | 3.55069 | Hydrophobic | Pi-Alkyl |
| :UNL1 - A:ALA467 | 4.88802 | Hydrophobic | Pi-Alkyl |
| :UNL1 - A:ILE441 | 4.96164 | Hydrophobic | Pi-Alkyl |
| :UNL1 - A:PRO444 | 5.42892 | Hydrophobic | Pi-Alkyl |
